# Supplementary material for: Polyketide synthase gene clusters in the ericoid mycorrhizal fungus Oidiodendron maius: genome-wide prediction, expression profiling, and functional characterization of knockout mutants
Source: Front Plant Sci. 2026 Sep 16;17:1924430. doi: 10.3389/fpls.2026.1924430 (PMC13623903; doi:10.3389/fpls.2026.1924430)
Supplement: Supplementary file 1 [file SupplementaryFile1.zip › Supplementary Materials/Supplementary Folder 1.zip!/Oidma1_AssemblyScaffolds_Repeatmasked/knownclusterblast/cluster2/jgi.p_Oidma1_109920_mibig_hits.html]

| MIBiG Protein | Description | MIBiG Cluster | MiBiG Product | % ID | % Coverage | BLAST Score | E-value |
| --- | --- | --- | --- | --- | --- | --- | --- |
| ABT18045.1 | delta-aminolevulinic\_acid\_dehydrotase/porphobilinogen\_synthase | BGC0000905 | Heme\_D1\_biosynthetic\_gene\_cluster | 43 | 94.0476190476 | 253 | 3.2e-67 |
| ABU41511.1 | HemB | BGC0000906 | Heme\_D1\_biosynthetic\_gene\_cluster | 43 | 94.0476190476 | 252 | 5.5e-67 |
